# Research of Planetary Gear Fault Diagnosis Based on Permutation Entropy of CEEMDAN and ANFIS

**DOI:** 10.3390/s18030782

**Published:** 2018-03-05

**Authors:** Moshen Kuai, Gang Cheng, Yusong Pang, Yong Li

**Affiliations:** 1 School of Mechatronic Engineering, China University of Mining and Technology, Xuzhou 221116, China; kuaimoshen2016@cumt.edu.cn (M.K.); liyong2015@cumt.edu.cn (Y.L.); 2 Faculty Mechanical, Maritime and Materials Engineering, Delft University of Technology, Delft 2628 CD, The Netherlands; Y.Pang@tudelft.nl

**Keywords:** planetary gear, fault diagnosis, CEEMDAN, permutation entropy, ANFIS

## Abstract

For planetary gear has the characteristics of small volume, light weight and large transmission ratio, it is widely used in high speed and high power mechanical system. Poor working conditions result in frequent failures of planetary gear. A method is proposed for diagnosing faults in planetary gear based on permutation entropy of Complete Ensemble Empirical Mode Decomposition with Adaptive Noise (CEEMDAN) Adaptive Neuro-fuzzy Inference System (ANFIS) in this paper. The original signal is decomposed into 6 intrinsic mode functions (IMF) and residual components by CEEMDAN. Since the IMF contains the main characteristic information of planetary gear faults, time complexity of IMFs are reflected by permutation entropies to quantify the fault features. The permutation entropies of each IMF component are defined as the input of ANFIS, and its parameters and membership functions are adaptively adjusted according to training samples. Finally, the fuzzy inference rules are determined, and the optimal ANFIS is obtained. The overall recognition rate of the test sample used for ANFIS is 90%, and the recognition rate of gear with one missing tooth is relatively high. The recognition rates of different fault gears based on the method can also achieve better results. Therefore, the proposed method can be applied to planetary gear fault diagnosis effectively.

## 1. Introduction

Gear transmission is a commonly used transmission method in mechanical equipment, in which planetary gear transmission has the advantages of large transmission ratio, strong carrying capacity, high transmission efficiency, etc., which is commonly used in the transmission system of mechanical equipment [1]. In planetary gear transmission process, affected by installation error, manufacture error and interference of environment, the planetary gear is not easy to be detected when it fails. Moreover, the vibration signals collected are affected by multi-gear meshing, which has nonlinear and non-stationary characteristics, increasing the difficulty of fault diagnosis and identification. At present, gear fault diagnosis and recognition can use physical model to analyze the gear state feature, and then generate health index can be got through data analysis. Finally, combined with geometric model, prediction of gear state can be completed [2]. The gear in the gearbox can also be diagnosed by the redundancy technology analytical redundancy [3].

The method of gear fault diagnosis proposed in this paper is based on the analysis of the vibration signals which are collected by an acceleration sensor. The fault diagnosis of planetary gear requires the signal processing of the vibration signal of planetary gear, so that the feature information in the signal can be obtained. Traditional signal processing method is based on Fourier Transform [4], but the disadvantage of this method is that the time domain signal and the frequency domain signal cannot be analyzed at the same time. To solve this problem, Wigner proposed Wigner distribution (WD) [5]. Though WD has better time-frequency resolution, the distribution cannot guarantee non negative, and it is not suitable for the analysis of multicomponent signals. EMD is a new time-frequency analysis method in signal processing, and it is more suitable for the analysis of nonlinear and non-stationary vibration signal compared with WD analysis methods [6]. And EMD has been widely used in the field of mechanical fault diagnosis [7,8]. But EMD itself has defects, including mode mixing, false mode and end point effect [9]. Therefore, Huang proposed the ensemble empirical mode decomposition (EEMD) on the basis of EMD [10]. This algorithm can effectively alleviate mode mixing of EMD by using Gaussian white noise which has uniform distribution in the frequency domain and zero mean. However, there are deficiencies in the decomposition process of EEMD. In the process of EEMD decomposition, the IMF components often carry residual noise, and the time of Gauss white noise added is different, which will bring difficulties to the final average operation [11]. In view of the reconstruction error of EEMD, complete ensemble empirical mode decomposition with adaptive noise (CEEMDAN) is proposed [12]. By adding the adaptive noise modal components to each stage of the EMD decomposition, and averaging the modal components after the decomposition, CEEMDAN is better than EEMD in decomposition ability, and effectively reduce the residual noise in the reconstructed signal. Since then, scholars have studied CEEMDAN, and achieved good results. Li [13] combined the EMD, EEMD, CEEMDAN and hybrid filtering methods to denoise the friction signal. The results showed that CEEMDAN can better preserve the signal features than EMD and EEMD. Humeau-Heurtier [14] proposed MCEEMDAN on the basis of CEEMDAN, which can accurately extract the data features of laser speckle contrast imaging (LSCI) with CEEMDAN, and effectively improve the understanding ability of image physiological information. Therefore, this paper chooses CEEMDAN to decompose the vibration signals of the planetary gear in each state, and the original signal is decomposed into a series of IMF which contain the fault features of planetary gear in different states.

The vibration signal is decomposed by CEEMDAN, which solves the problem that the traditional decomposition method cannot effectively decompose the non-stationary and nonlinear vibration signal. However, the fault feature information in the IMF after CEEMDAN decomposition is not obvious and needs to be quantified. At present, the commonly used methods of quantifying fault information are fractal dimension, sample entropy and permutation entropy [15,16,17]. And the permutation entropy is a random detection method of time series proposed by Bandt [18], which has the advantages of simple design, strong anti-noise ability and fast running speed [19]. It can be used to quantify the fault features of rotating mechanical vibration signal. Cheng [20] proposed the multi-scale permutation entropy based on permutation entropy to complete quantization of fault features of the bearing signal, and then refined feature vectors with Laplacian operator. Finally, classification and recognition were realized by the support vector machine. So, permutation entropy can be used to extract fault features of different states of sun gears.

After the mechanical vibration signal is decomposed and quantized, the fault feature vectors in the signal can be extracted. The extracted feature vectors are identified by the identification model to determine the fault types of sun gears. At present, the identification algorithms commonly used in mechanical equipment fault diagnosis are fuzzy algorithm and neural network algorithm [21], but both of the two algorithms have their own limitations. The fuzzy algorithm has a strong ability of decision-making and reasoning, but the self-learning and adaptive ability is poor; the neural network algorithm has good adaptability and nonlinear learning ability, but existing experience and knowledge cannot be effectively expressed. Adaptive Neural-Network-Based Fuzzy Interference System (ANFIS) is proposed by Jang [22] based on Takagi-Sugeno (T-S) which can be used in fault detection filter design and sensor faults estimation [23,24], integrating the advantages of both fuzzy algorithm and neural network algorithm [25]. By incorporating the neural network into each step of the fuzzy reasoning process, the fuzzy rules and membership function parameters in the model can be adjusted adaptively, which makes the model have high prediction accuracy, and the algorithm can be completed under the condition of less samples. In biomedicine, ANFIS has made some progress. Uğuz [26] used discrete wavelet transform to decompose the heart sound signal into several sub-bands, and quantified it by Shannon entropy. Finally, detection of heart valve disease is completed by using ANFIS to classify the heart sound signal. In the mechanical equipment fault diagnosis, ANFIS has also achieved good results. Cheng [27] combined LMD with fuzzy entropy to extract features of gears, and then used ANFIS classifier to identify the fault categories of gears. The recognition results showed that ANFIS has higher accuracy and better practicability.

In this paper, a fault diagnosis method based on CEEMDAN-permutation entropy ANFIS planet gear is proposed to solve the problem that the state of the planetary gear fault is difficult to identify under the condition of constant load. It is proved by the experiment that this method can realize the fault diagnosis of the planetary gear under the condition of constant load and the method is mainly based on application of sensors. This paper is structured as follows. The second part establishes a mathematical model for fault diagnosis based on permutation entropy of CEEMDAN and ANFIS. The third part presents experiments using DDS comprehensive mechanical fault simulation bench and equipment which are acceleration sensors for collecting vibration signals of planetary gear. The forth part carries on the experiment analysis. The collected vibration signals are decomposed into a series of IMFs by CEEMDAN, and the permutation entropy of each IMF is extracted as the fault feature. Label fault features and make them as inputs of ANFIS classifier to diagnose and identify fault models through the corresponding outputs. The last part puts forward the conclusion of this paper.

## 2. Building Model

### 2.1. CEEMDAN Signal Decomposition Method

EEMD decomposition is made improvement based on the EMD decomposition, using the mean scale characteristics of white noise to make the signal continuous in each scale, so that the distribution of extreme points in the signal change, which effectively solves the problem, mode mixing, of EMD.

Although EEMD can effectively alleviate mode mixing, a problem of EMD, EEMD cannot maintain the completeness of EMD, and it contains residual noise in each IMF component and residual component of its decomposition, which affects the accuracy of reconstructing the original signal. Therefore, the CEEMDAN algorithm is proposed. The multiple EMD decomposition is performed on the adaptive noise component added to each stage, and IMF is calculated by averaging the results. Thus completeness of EMD is maintained, and the problems which are model mixing problem and reconstruction errors caused by the low efficiency are solved.

To better describe CEEMDAN algorithm, define an operator $E_{k}(\cdot )$, its function is to solve the *k*th modal component ${\mathrm{IMF}}_{k}$ of EMD decomposition. Let $w^{i}$ be the white noise satisfying distribution of N(0, 1), and $\varepsilon_{k}$ is the amplitude coefficient of white noise added for the *K*th time. The decomposition process for the specific CEEMDAN is shown below:

(1) The white noise $X(t)+\varepsilon_{0}\omega^{i}(t)$ is added to the original signal, and *I*th EMD decomposition is performed. Then complete the average operation on the result to get ${\mathrm{IMF}}_{1}$.
  (1) $${\mathrm{IMF}}_{1}=\frac{1}{I}\sum_{i=1}^{1}E_{1}(X(t)+\varepsilon_{0}\omega^{i}(t))$$
(2) The first stage residual component can be calculated.
  (2) $$r_{1}(t)=X(t)-{\mathrm{IMF}}_{1}$$
  The white noise $r_{1}(t)+\varepsilon_{1}E_{1}(\omega^{i}(t))$, i=1,2,…,I is added to the first stage residual component, and the EMD is performed. Then ${\mathrm{IMF}}_{2}$ can be calculated with the mean value of the first IMF.
  (3) $${\mathrm{IMF}}_{2}=\frac{1}{I}\sum_{i=1}^{1}E_{1}(r_{1}(t)+\varepsilon_{1}E_{1}(\omega^{i}(t)))$$
  For *k* = 1, 2, …, *K*, the *K*th residual component can be calculated.
  (4) $$r_{k}\left(t\right)=r_{k-1}\left(t\right)-{\mathrm{IMF}}_{k}$$
(3) Adding white noise $r_{1}(t)+\varepsilon_{1}E_{1}(\omega^{i}(t))$, i=1,2,…,I to the *k*th residual component and performing EMD decomposition. Then ${\mathrm{IMF}}_{k+1}$ can be calculated with the mean value of the first IMF.
  (5) $${\mathrm{IMF}}_{k+1}=\frac{1}{I}\sum_{i=1}^{1}E_{1}(r_{k}(t)+\varepsilon_{k}E_{k}(\omega^{i}(t)))$$
(4) Repeat Step (4) and Step (5) until the value of residual component is less than two extremes, then the decomposition stops. Eventually the residual variable is obtained.
  (6) $$r(t)=X(t)-\sum_{k=1}^{K}{\mathrm{IMF}}_{k}$$
  where *K* is the total number of modes in the decomposition process, the reconstructed signal can be expressed as follows:
  (7) $$X(t)=r(t)+\sum_{k=1}^{K}{\mathrm{IMF}}_{k}$$

### 2.2. Permutation Entropy

Entropy is a quantitative tool to describe the complexity of a system, and entropy varies with the state of the system. Permutation entropy is a kind of random time sequence detection method, and can reflect the one-dimensional time series complexity, which has advantage of simple design, strong anti-noise ability, better robustness and is suitable for feature extraction of nonlinear data. The following is the specific principle of permutation entropy algorithm.

Assuming the time series {X(i),i=1,2,…,N} with length *N*, the phase space reconstruction is carried out as follows:

(8) $$\left[\begin{array}{cccc} x(1) & x(1+\tau ) & \ldots & x(1+(m-1)\tau )\\ x(2) & x(2+\tau ) & \ldots & x(2+(m-1)\tau )\\ x(3) & x(3+\tau ) & \ldots & x(3+(m-1)\tau )\\ \ldots & \ldots & \ldots & \ldots \\ x(K) & x(K+\tau ) & \ldots & x(K+(m-1)\tau ) \end{array}\right]$$

where m and τ represent the embedded dimension and the delay time, respectively, and each row vector in the matrix represents a reconstructed component. The elements x(j), x(j+τ), …, x(j+(m−1)τ) of each row vector in the matrix are rearranged in ascending order.

(9) $$x(i+(j_{1}-1)\tau )\leq x(i+(j_{2}-1)\tau )\leq \ldots \leq x(i+(j_{m}-1)\tau )$$

Thereby, a new set of row vectors X(i) is obtained.

If there are two elements in the reconstructed row vector equal to each other, such as $x(i+(j_{1}-1)\tau )=x(i+(j_{2}-1)\tau )$, they should be sorted by the column size of $x(i+(j_{1}-1)\tau )$ and $x(i+(j_{2}-1)\tau )$($j_{1}<j_{2}$), as follows:

(10) $$x(i+(j_{1}-1)\tau )\leq x(i+(j_{2}-1)\tau )$$

Therefore, every row vector in the reconstructed matrix can be expressed as a set of symbolic sequences for any time series.

(11) $$S(l)=\{j_{1},j_{2},\ldots ,j_{m}\text{\}}$$

In the formula, *l* = 1, 2, …, *k*, k≤m!, S(l) composed of m elements has m! arrangements, so S(l) can have m! symbol sequence. If the probabilities of *k* kinds of different symbol sequences are $P_{1}$, $P_{2}$, …, $P_{k}$, $\sum_{s=1}^{k}P_{s}=1$, respectively. So the permutation entropy $H_{p}$ of time series {X(i),i=1,2,…,N} can be defined as the form of information entropy:

(12) $$H_{p}(d)=-\sum_{j=1}^{k}P_{j}\ln(P_{j})$$

Normalizing $H_{p}(d)$, as follows:

(13) $$H_{p}(d)=H_{p}(d)/\ln(m!)$$

The value range of $H_{p}(d)$ is [0,1], and the size of the permutation entropy reflects the randomness of the time series. The larger the permutation entropy is, the greater the randomness of the time series. The smaller the permutation entropy is, the more regular the time series.

According to the principle of permutation entropy, the embedded dimension *m* and the time delay τ are two key parameters that determine the value of permutation entropy, and the value of embedding dimension was proposed between 3 and 7. If the value of embedding dimension is too small, algorithm will not effectively reflect the dynamic mutation of time series, which makes permutation entropy lose effectiveness and practical significance. If the value of embedding dimension is too large, embedded dimension value will not reflect the subtle change accurately, which can affect computing efficiency of permutation entropy. Yan [28] found that when m=6 and τ=3, permutation entropy can better reflect the subtle changes of mechanical system. Therefore, this paper permutation entropy embedding dimension m=6 and time delay τ=3.

### 2.3. Adaptive Neuro-Fuzzy Inference System

**Figure 1:**
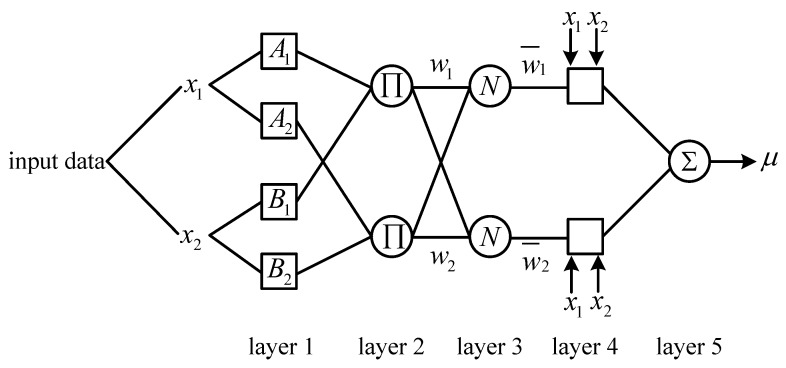
ANFIS structure.

The typical ANFIS structure consists of a two-input-single-output 5-layer network. As shown in Figure 1, the squares and the circles represent the nodes with adjustable parameters and non-adjustable parameters, respectively.

(1) Layer 1 is input layer which is composed of square nodes, and the membership degree of the output fuzzy set corresponding to each input is calculated by blurring the input quantity. The transfer function transmitted from the first layer nodes to the second layer nodes can be expressed as follows:
  (14) $$\left\{ \begin{array}{l} O_{i,j}=\mu_{A_{i}}(x_{1}),i=1,2\\ O_{i,j}=\mu_{B_{i-2}}(x_{2}),i=3,4 \end{array}\right.$$
  where $x_{1}$ and $x_{2}$ are two inputs of ANFIS, $\mu_{A_{i}}$ and $\mu_{B_{i-2}}$, which often use Gauss function, are membership functions of fuzzy sets.
(2) Layer 2 is rule operation layer which is composed of round nodes. Each node represents one rule. The fitness of each rule is obtained by performing product operation, which is expressed as follows:
  (15) $$O_{2,j}=w_{i}=\mu_{A_{i}}(x_{1})\mu_{B_{i}}(x_{2})\;i=1,2$$
(3) Layer 3 is normalized layer which is composed of circular nodes, whose function is to the normalize fitness fuzzy rules.
  (16) $$O_{3,j}={\bar{w}}_{i}=\frac{w_{i}}{w_{1}+w_{2}}\;i=1,2$$
  where $w_{i}$ is the fitness of the *i*th rule.
(4) Layer 4 is rule output layer which is composed of square nodes. Each node’s transfer function is a linear function, whose role is to calculate the output of all fuzzy rules, expressed as follows:
  (17) $$O_{4,j}={\bar{w}}_{i}f_{i}={\bar{w}}_{i}(p_{i}x_{1}+q_{i}x_{2}+r_{i})\;i=1,2$$
  where ${\bar{w}}_{i}$ represents the output value of the rule layer, and the set composed of $p_{i}$, $q_{i}$, $r_{i}$ is the conclusion parameter set of the regular layer.
(5) Layer 5 is output layer which is composed of round nodes, whose role is to calculate the sum of all outputs. It can be expressed as follows:
  (18) $$O_{5,i}=\sum_{i}\bar{w_{i}}f_{i}=\frac{\sum_{i}w_{i}f_{i}}{\sum_{i}w_{i}}$$
  The essence of fault diagnosis research using ANFIS is to adjust the premise parameter and conclusion parameter of the model constantly. The correction method generally includes BP algorithm and hybrid algorithm. However, in the practical application of the fault diagnosis, both hybrid algorithm and BP algorithm have slow training speed, which can make algorithms easily fall into the local minimum. Therefore, this study overcomes slow convergence which BP algorithm usually has by using numerical optimization technique and Levenberg-Marquart algorithm.

## 3. Experimental Equipment and Data Acquisition

**Figure 2:**
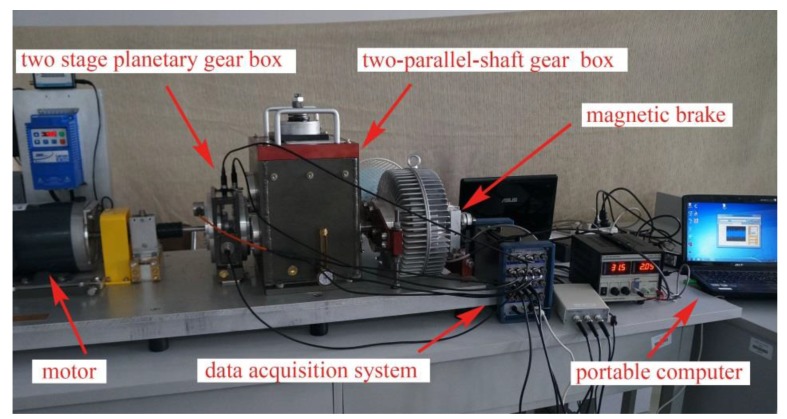
Fault experiment for planetary gear.

**Figure 3:**
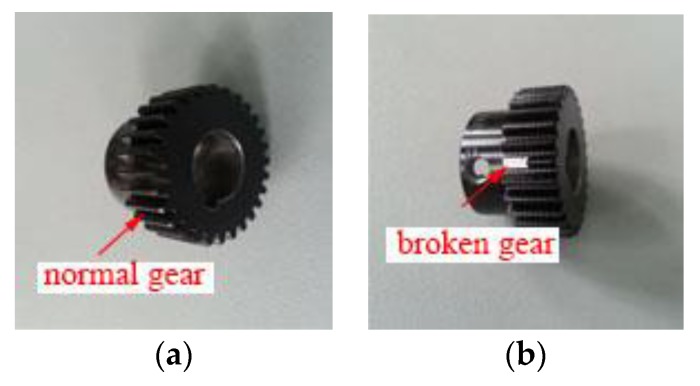

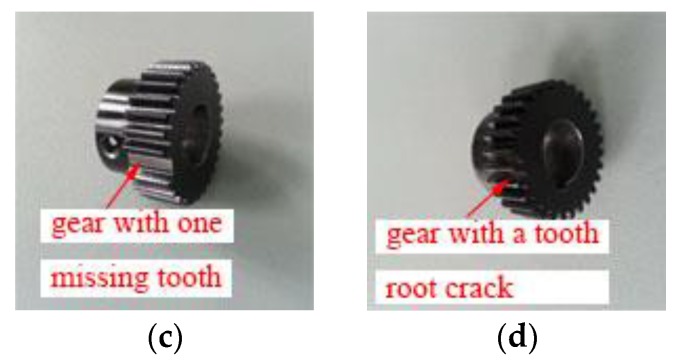
Four types of gears, (**a**) normal gear, (**b**) broken gear, (**c**) gear with one missing tooth, (**d**) gear with a tooth root crack.

**Figure 4:**
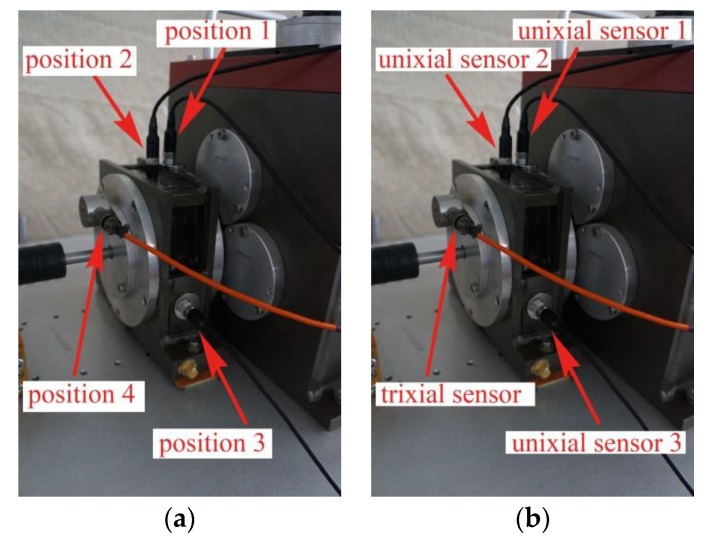
Layout mode of acceleration sensor (**a**) location of acceleration sensor, (**b**) type of acceleration sensor.

**Figure 5:**
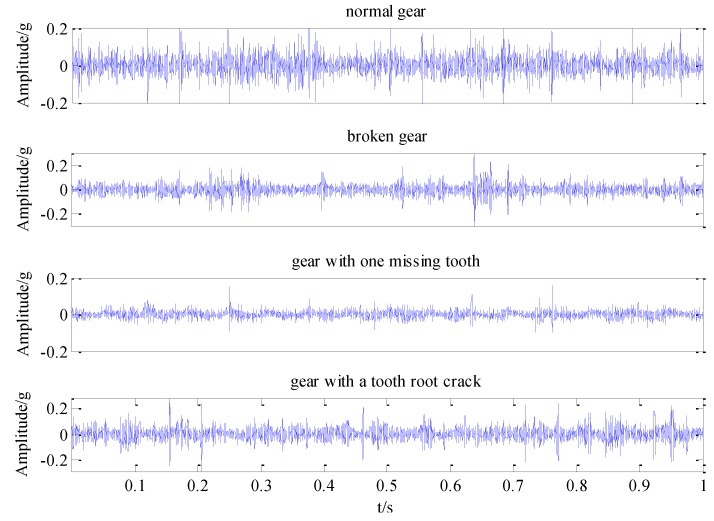
Vibration signals of four different states of sun gears in constant load condition.

In order to verify the feasibility and effectiveness of the proposed gear fault diagnosis method based on permutation entropy of CEEMDAN and ANFIS, the experiment uses the machinery fault simulator made by Spectra Quest, an American company. Experiment table, as shown in Figure 2, consists of the following systems: motor, two stage planetary gear box, two-parallel-shaft gearbox consisting of a rolling bearing or two parallel shafts, bearing load, magnetic brake, vibration sensor, signal acquisition system and portable computer. The test bench contains configuration of the powertrain, which is suitable for study based on diagnostic technology, lubrication conditions, gear box dynamics and health monitoring of abrasive particle analysis, noise characteristics and vibration characteristics. So the experiment table can simulate four fault states which are normal gear, broken gear, gear with one missing tooth and gear with a tooth root crack, as shown in Figure 3. And the basic parameters of the second-stage planetary gear are shown in Table 1. Because of the requirement of load and load change, the setting of gear load can be accomplished by programming. The vibration signal is detected by the 608A11PCB acceleration sensor produced by IMI. The layout mode of acceleration sensor is shown in Figure 4. The main factor affecting the signal acquisition of planetary gear is the installation position of the sensor. In this paper, the main study of the two stage planetary gear fault, and the main selection of placement of sensors to collect planetary gear’s vibration signals are position 1, position 2, position 3 and position 4. From the structure of the two stage planetary gear, the position 1 is more accurate for collecting the vibration signals of the sun gear, and it is found that vibration information is mainly concentrated on the vertical plane, so the single axis sensor installed at position 1 is selected for signal acquisition of the sun gear. The sensor has a detection frequency range of 500~10,000 Hz and a resolution of 350 g. The vibration signal detected by the acceleration sensor is accurately collected by a computer connected to the bench. In the process of gear transmission, high speed rotating sun gear meshes with multiple planetary gears at the same time, which is liable to fail. Therefore, this paper takes the sun gear damage of planetary gear as an example, and performs fault diagnosis on four states of sun gear, normal, broken teeth, missing teeth and tooth root. In the process of experiment, the output frequency of the motor is set as 40 Hz, and the operating condition is constant load. And the load controlled by a programmable brake component is set as 13.5 Nm. In the experiments, the sampling frequency is set as 13,107.2 Hz. The four kinds of vibration signals of the sun gear under constant load are collected and shown in Figure 5.

## 4. Experimental Analysis

**Figure 6:**
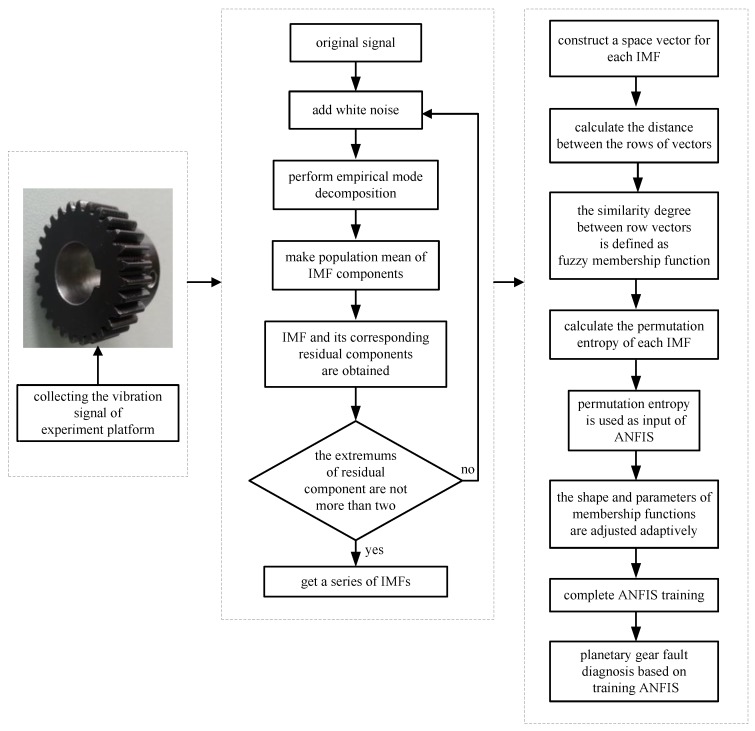
Experimental analysis flowchart.

**Figure 7:**
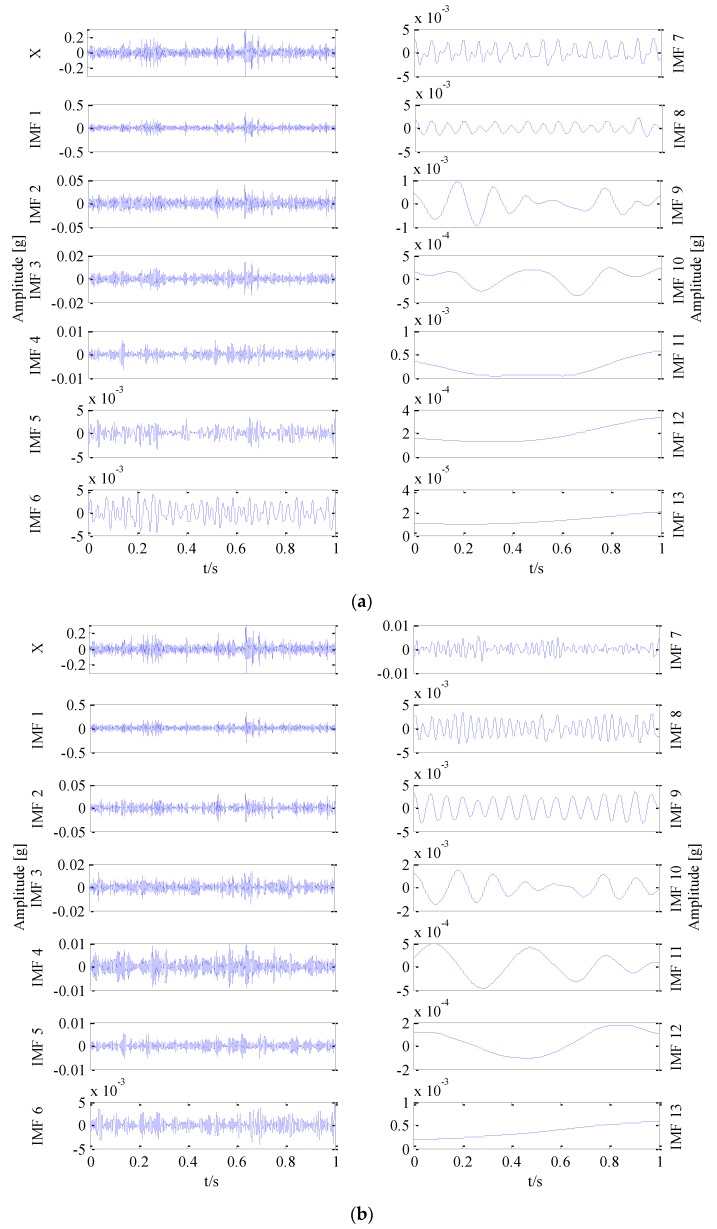
Decomposition of broken gear, (**a**) EEMD decomposition of broken gear, (**b**) CEEMDAN decomposition of broken gear.

The experimental analysis flowchart of the fault diagnosis method based on Permutation Entropy of CEEMDAN and ANFIS is proposed and shown in Figure 6. The vibration signals of the sun gear are collected in four different states, such as normal gear, broken gear, gear with one missing tooth and gear with a tooth root crack, as shown in Figure 5. In the condition of constant load of motor, the vibration signals of four states are sampled 50 times respectively, and 200 sets of original samples are obtained. Every sample signal is decomposed by CEEMDAN to obtain a series of IMF components, the signal sample length is 5120, the average number of times is 100, and the standard deviation of the added noise is 0.15 times of the original signal. Due to limited space, taking the vibration signal of broken gear as an example to show the decomposition process of CEEMDAN and EEMD in Figure 7, and it can be found that the decomposition result of CEEMDAN is superior to that of EEMD. For EEMD, it is obvious that there is the phenomenon of modal aliasing in IMF6 and IMF8 which will affect the extraction of fault information. The non-stationary of four different states of sun gears’ the vibration signals makes CEEMDAN decompose many IMF components. In order to improve the recognition accuracy of the prediction model, using permutation entropy algorithm to reflect complexity of each IMF component, which completes the quantitative features of fault information.

**Figure 8:**
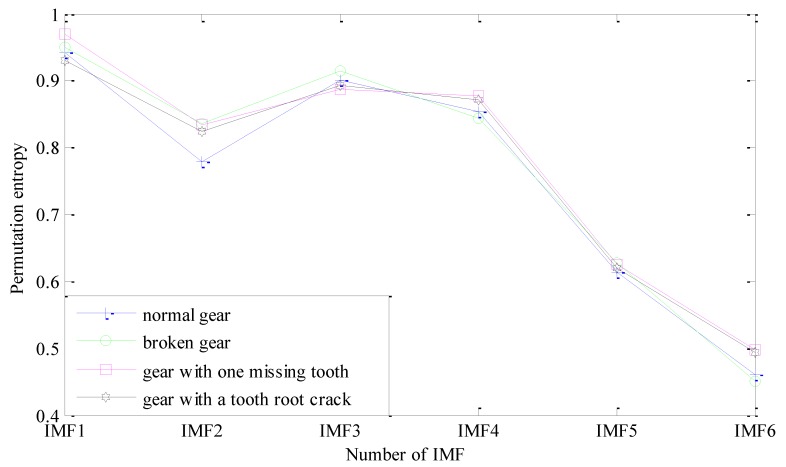
Permutation entropies of each IMF component of four different states of sun gears.

A series of IMF components decomposed by CEEMDAN contain fault information of the sun gear vibration signal in different time series, and the fault information is generally concentrated in the relatively high frequency band of vibration signal. Therefore, the first 6 IMFs are selected as the analysis objects to extract the fault feature of the sun gear. For the sake of length, this paper only lists permutation entropy values of the first three sets of samples of the sun gear in four different states, as is shown in Table 2. For the fault features of gears are mainly concentrated in the high frequency sections, the first six IMF components which contain the main features are selected as the research objects. As shown in Figure 8. Through calculation of permutation entropy of CEEMDAN, it can be clearly see that IMF1~IMF4 and IMF6 can represent main fault features of four different states of sun gears, and the fault features are more obvious, which can make the recognition model more quickly and accurately identify fault models of sun gears. Therefore IMF1~IMF4 and IMF6 can be chosen as the fault features of four different states of sun gears.

**Figure 9:**
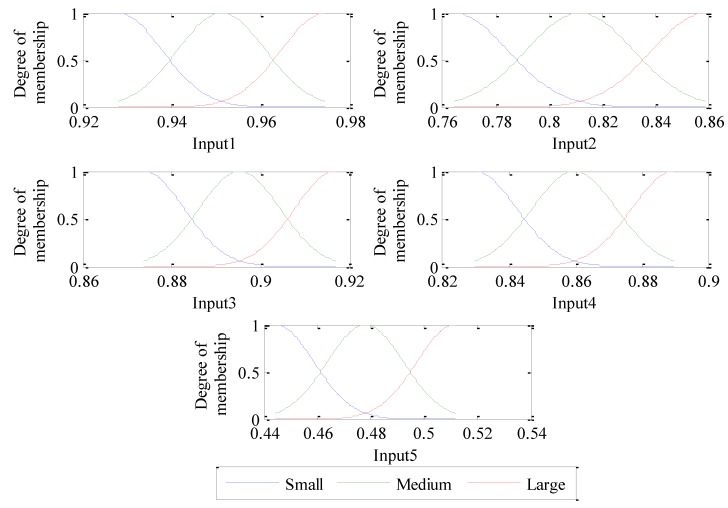
Initial membership functions of each input of ANFIS.

Since the above analysis is only based on a small amount of samples analysis, which is random and cannot verify the validity of fault features extraction for fault classification and identification. In this regard, adaptive neuro-fuzzy inference system is constructed, and 50 sets of sample data are collected from four different states of sun gears. Thus, the four different states of sun gears have 200 samples. The first 30 groups are used as training samples, and the left 20 groups are used as testing samples. Using CEEMDAN decomposition algorithm to get the IMF components, and permutation entropy is calculated to get feature vectors respectively. These feature vectors with obvious fault features are used to train ANFIS, and the same method is used to identify testing samples. The ANFIS model has 5 inputs, each input is configured with three Gauss membership functions, and inputs are divided into three levels which are large, medium, and small. Figure 9 shows the initial ANFIS model of each input membership function.

**Figure 10:**
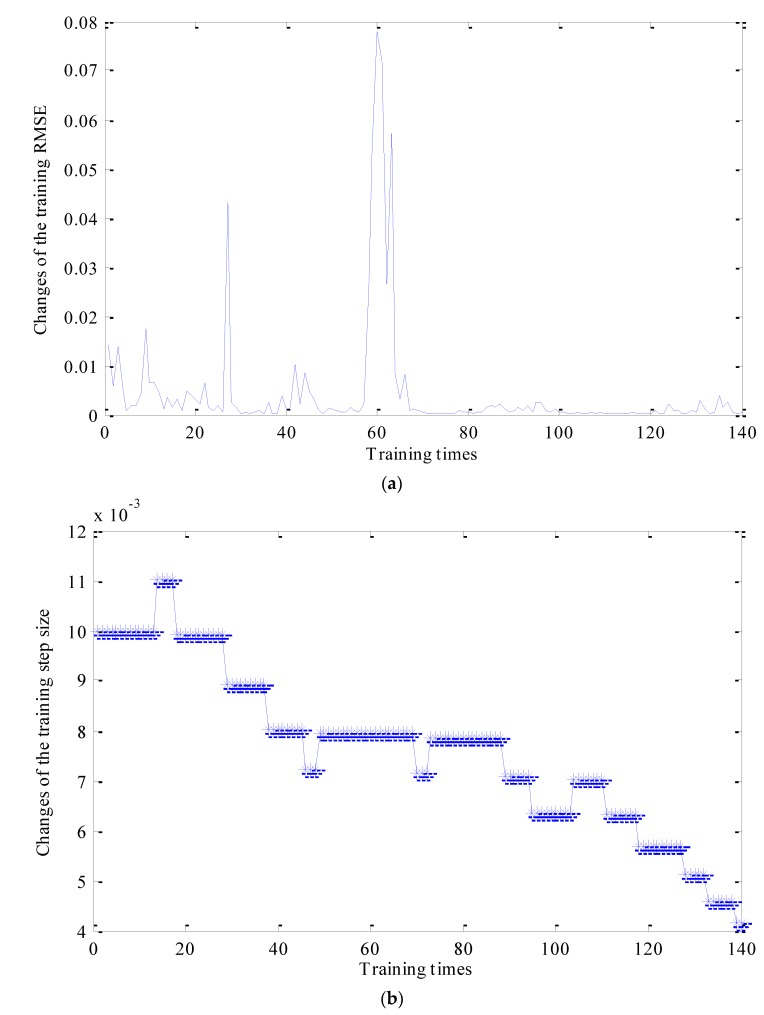
Changes of the training RMSE and the training step size during the training process: (**a**) the changes of training RMSE; (**b**) the changes of the training step size.

The outputs of ANFIS are states of sun gears. Therefore, in order to train the ANFIS model, four different states of sun gears are labeled with different numbers, such as: normal gear-1, broken gear-2, gear with one missing tooth-3, gear with a tooth root crack-4. ANFIS model parameters and membership functions are adjusted adaptively according to the training samples. The root mean square error (RMSE) is used to evaluate the training process. The training process is controlled by setting RMSE and training time. In the process of training, following methods are adopted for adjusting training steps: When the RMSE reaches a local maximum, the training step remains stable; When RMSE stabilizes at around 0.002, the training step decreases four times in a row. The changes the training step size is shown in Figure 10a, and the changes of training RMSE is shown in Figure 10b.

**Figure 11:**
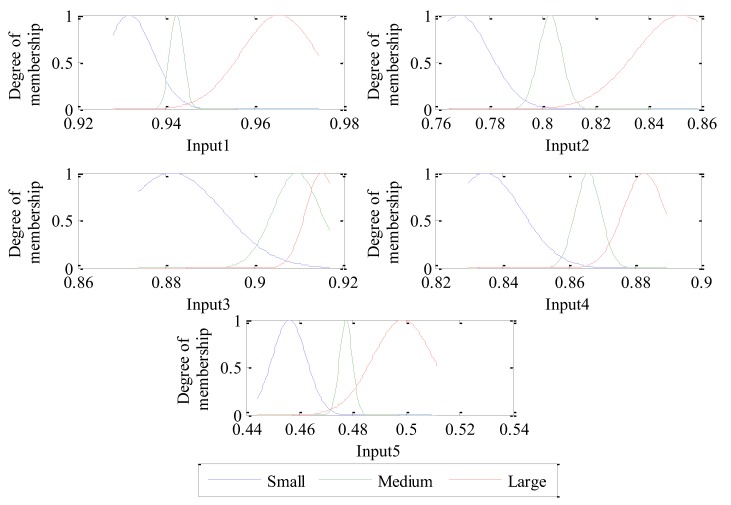
Final adjusted membership functions of each input of the trained ANFIS.

Figure 10a shows that the training RMSE dropped to about 0.002 after 140 training times. When the training process is completed, the parameters and shapes corresponding to the membership functions of each input are adjusted to the most suitable values. The final adjusted membership function corresponding to each input is shown in Figure 11. Compared with Figure 9 and Figure 11, the membership functions of inputs 2 and 4 change slightly, and the membership functions of inputs 1, 3 and 5 vary observably.

**Figure 12:**
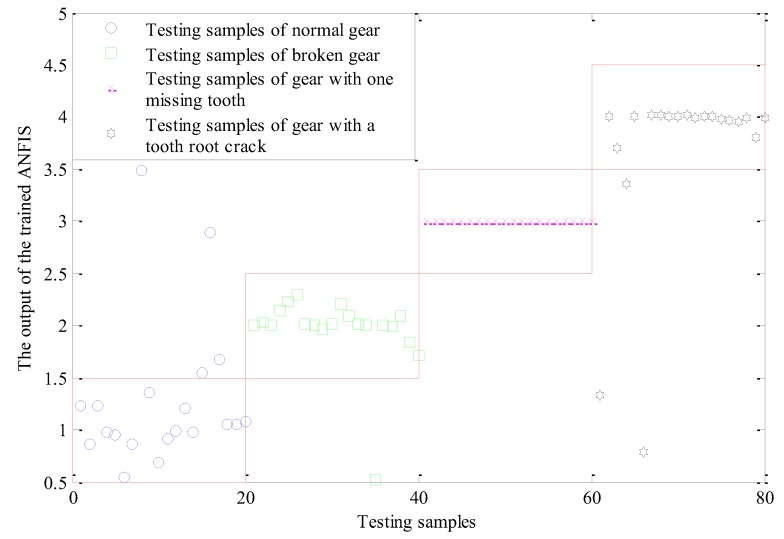
Output of the trained ANFIS.

The effectiveness of the trained ANFIS model in determining states of sun gears are demonstrated by using testing samples to verify the trained ANFIS model. The permutation entropy of each IMF component of the testing samples is used as input of the training ANFIS model, corresponding to the output is shown in Figure 12. Different states of sun gears need different labels in the training process to distinguish the different sun gear states, namely the normal gear-1, the broken gear-2, gear with one missing tooth-3, gear with a tooth root crack-4. When the testing samples are used as inputs, sun gear states are identified and diagnosed according to the outputs of the trained ANFIS model. If the output in the interval [0.5, 1.5], it should be normal gear. If the output in the interval [1.5, 2.5], it should be broken gear. If the output in the interval [2.5, 3.5], it should be gear with one missing tooth. If the output in the interval [3.5, 4.5], it should be gear with a tooth root crack. If the output exceeds the range of [0.5, 5.5], it is unable to determine the state of sun gear. Figure 12 shows that the trained ANFIS model has the best performance in fault recognition of gear with one missing tooth, and the fault recognition rate is 100%. The fault recognition rate of broken gear is 95%, and the recognition rate of normal gear and gear with a tooth root crack is 80% and 85% respectively. The overall fault recognition rate is 90%, showing that the performance of ANFIS model after training has better performance. 

The BP error convergence factor is set to 0.1, and the error target value is 0.00004, meanwhile the number of neurons is set to 100. By comparing two kinds of identification results of planetary gear fault diagnosis, as is shown in the Table 3, planetary gear fault diagnosis based on permutation entropy of CEEMDAN and ANFIS has obvious advantages. Therefore, the proposed planetary gear fault diagnosis method can extract fault features, and more accurately identify sun gear states, which is an effective method to diagnose the planetary gear fault.

## 5. Conclusions

This paper presents a planetary gear fault diagnosis method based on permutation entropy of CEEMAN and ANFIS. The planetary gear vibration signal is decomposed with CEEMAN into a series of IMF components. The time complexity of each IMF component is evaluated by permutation entropy, so that the fault features of sun gear in different states are extracted. Then, the feature vectors of the sun gears’ faults in different states are defined as inputs of the ANFIS model, and the number of labeled sun gear in four different states are defined as outputs of the ANFIS model. The initial membership function of each input is set as Gauss function, and the training process of ANFIS is controlled by RMSE and training time. In the process of training ANFIS, the membership and shape of each input and other ANFIS parameters can be adjusted according to the training samples, and finally the optimal ANFIS model is obtained. The test samples are evaluated and identified by the optimal ANFIS model, and test result is that fault recognition rate of gear with one missing tooth is 100%, fault recognition rate of broken gear is 95%, and fault recognition rate of normal gear and gear with a tooth root crack are 85% and 80%, respectively. The overall fault recognition rate is 90%, showing that ANFIS model perform well after training. By comparing two kinds of identification results of planetary gear fault diagnosis, planetary gear fault diagnosis based on permutation entropy of CEEMDAN and BP and planetary gear fault diagnosis based on permutation entropy of CEEMDAN and ANFIS, planetary gear fault diagnosis based on permutation entropy of CEEMDAN and ANFIS has obvious advantages. The results illustrate that the proposed planetary gear fault diagnosis method based on permutation entropy of CEEMDAN and ANFIS can accurately extract fault features generated from four different states of sun gears, and realize fault diagnosis of sun gears well.

## Figures and Tables

**Table 1:** Basic parameters of the second-stage planetary gear.

Parameter	Transmission Ratio	Pressure Angle	Material	Module	Number of Sun Gear	Number of Teeth on the Sun Gear	Number of Inner Ring Gear
Value	4.57	20°	S45C	1	28	36	100

**Table 2:** Permutation entropies of IMFs.

Type	IMF1	IMF2	IMF3	IMF4	IMF5	IMF6
Normal gear	0.9422	0.7792	0.9012	0.8525	0.6132	0.4595
	0.9527	0.7924	0.9119	0.8679	0.6170	0.4684
	0.9513	0.7930	0.9029	0.8603	0.6320	0.4837
Broken gear	0.9500	0.8356	0.9151	0.8431	0.6277	0.4497
	0.9517	0.8199	0.9109	0.8541	0.6045	0.4583
	0.9517	0.8276	0.9132	0.8507	0.6069	0.4748
Gear with one missing tooth	0.9687	0.8366	0.8859	0.8774	0.6258	0.4968
	0.9688	0.8379	0.8882	0.8784	0.6233	0.4889
	0.9694	0.8319	0.8850	0.8855	0.6130	0.4942
Gear with a tooth root crack	0.9286	0.8156	0.8954	0.8734	0.6245	0.4794
	0.9379	0.8109	0.9009	0.8767	0.6411	0.4885
	0.9328	0.8161	0.8972	0.8707	0.6170	0.4776

**Table 3:** Recognition rate of permutation entropy of CEEMDAN and BP and permutation entropy of CEEMDAN and ANFIS.

Fault Modes	Normal Gear	Broken Gear	Gear with One Missing Tooth	Gear with a Tooth Root	Overall
The number of trained samples	30	30	30	30	120
The number of test samples	20	20	20	20	80
Permutation entropy of CEEMDAN and BP Accuracy (%)	85	95	100	60	85
Permutation entropy of CEEMDAN and ANFIS Accuracy (%)	80	95	100	85	90

## References

[1] Lei Y.G., Tang W., Kong D.T., Lin J. (2014). Vibration signal simulation and fault diagnosis of planetary gearboxes based on transmission mechanism analysis. J. Mech. Eng..

[2] Djeziri M.A., Benmoussa S., Sanchez R. (2017). Hybrid method for remaining useful life prediction in wind turbine systems. Renew. Energy.

[3] Praveenkumar T., Sabhrish B., Saimurugan M., Ramachandran K.I. (2018). Pattern recognition based on-line vibration monitoring system for fault diagnosis of automobile gearbox. Measurement.

[4] Prusa Z., Balazs P., Sondergaard P.L. (2017). A noniterative method for reconstruction of phase from STFT magnitude. IEEE/ACM Trans. Audio Speech Lang. Process..

[5] Wigner E.P. (1932). On the quantum correction for thermodynamic equilibrium. Phys. Rev..

[6] Ananou B., Ouladsine M., Pinaton P., Nguyen L., Djeziri M., Djohor F. (2016). Fault prognosis for batch production based on percentile measure and gamma process. J. Process Control.

[7] Cheng G., Cheng Y.L., Shen L.H., Qiu J.B., Zhang S. (2013). Gear fault identification based on Hilbert-Huang transform and SOM neural network. Measurement.

[8] Liu D., Zeng H.T., Xiao Z.H., Peng L.H., Malik O.P. (2017). Fault diagnosis of rotor using EMD thresholding-based de-noising combined with probabilistic neural network. J. Vibroeng..

[9] Guo T., Deng Z.M. (2017). An improved EMD method based on the multi-objective optimization and its application to fault feature extraction of rolling bearing. Appl. Acoust..

[10] Wu Z.H., Huang N.E. (2005). Ensemble empirical mode decomposition: A noise-assisted data analysis method. Adv. Adapt. Data Anal..

[11] Choudhary V., Dhami S.S., Pabla B.S. (2017). Non-contact incipient fault diagnosis method of fixed-axis gearbox based on CEEMDAN. R. Soc. Open Sci..

[12] Torres M.E., Colominas M.A., Schlotthauer G., Flandrin P. (2011). A complete ensemble empirical mode decomposition with adaptive noise. Brain Res. Bull..

[13] Li C.W., Zhan L.W., Shen L.Q. (2015). Friction signal denoising using complete ensemble EMD with adaptive noise and mutual information. Entropy.

[14] Humeau-Heurtier A., Mahe G., Abraham P. (2015). Multi-dimensional complete ensemble empirical mode decomposition with adaptive noise applied to laser speckle contrast images. IEEE Trans. Med. Imaging.

[15] Reljin N., Reyes B.A., Chon K.H. (2015). Tidal volume estimation using the blanket fractal dimension of the tracheal sounds acquired by smartphone. Sensors.

[16] Chen J.Y., Zhou D., Lyu C., Lu C. (2017). An integrated method based on CEEMD-SampEn and the correlation analysis algorithm for the fault diagnosis of a gearbox under different working conditions. Mech. Syst. Signal Process..

[17] Shi Z.L., Song W.Q., Taheri S. (2016). Improved LMD, permutation entropy and optimized K-Means to fault diagnosis for roller bearings. Entropy.

[18] Bandt C., Pompe B. (2002). Permutation entropy: A natural complexity measure for time series. Phys. Rev. Lett..

[19] Zhang X.Y., Liang Y.T., Zhou J.Z., Zang Y. (2015). A novel bearing fault diagnosis model integrated permutation entropy, ensemble empirical mode decomposition and optimized SVM. Measurement.

[20] Zhen J.D., Cheng J.S., Yang Y. (2014). Multiscale permutation entropy based rolling bearing fault diagnosis. Shock Vib..

[21] Jiang P., Hu Z.X., Liu J., Yu S.N., Wu F. (2016). Fault diagnosis based on chemical sensor data with an active deep neural network. Sensors.

[22] Jang J.S.R. (1993). ANFIS: Adaptive-network-based fuzzy inference systems. IEEE Trans. Syst. Man Cybern..

[23] Chilbani A., Chadli M., Shi P., Braiek N.B. (2017). Fuzzy Fault Detection Filter Design for T-S Fuzzy Systems in Finite Frequency Domain. IEEE Trans. Fuzzy Syst..

[24] Youssef T., Chadli M., Karimi H.R., Wang R. (2017). Actuator and sensor faults estimation based on proportional integral observer for TS fuzzy model. J. Frankl. Inst..

[25] Pradhan B. (2013). A comparative study on the predictive ability of the decision tree, support vector machine and neuro-fuzzy models in landslide susceptibility mapping using GIS. Comput. Geosci..

[26] Uğuz H. (2012). Adaptive neuro-fuzzy inference system for diagnosis of the heart valve diseases using wavelet transform with entropy. Neural Comput. Appl..

[27] Cheng G., Chen X.H., Li H.Y. (2016). Study on planetary gear fault diagnosis based on entropy feature fusion of ensemble empirical mode decomposition. Measurement.

[28] Yan R.Q., Liu Y.B., Gao R.X. (2012). Permutation entropy: A nonlinear statistical measure for status characterization of rotary machines. Mech. Syst. Signal Process..

